# Distributed Artificial Intelligence for IoT Security: A Structured Review

**DOI:** 10.3390/s26154802

**Published:** 2026-07-28

**Authors:** Sabina Szymoniak, Mariusz Kubanek

**Affiliations:** Department of Computer Science, Czestochowa University of Technology, 42-201 Częstochowa, Poland; mariusz.kubanek@icis.pcz.pl

**Keywords:** Internet of Things, security, Distributed Artificial Intelligence

## Abstract

The expansion of the Internet of Things (IoT) has increased the complexity of securing distributed systems against growing threats to data security, privacy, and reliability. Conventional centralised cybersecurity methods are often insufficient for environments characterised by scale, heterogeneity, and dynamic behaviour. This paper presents a structured review of Distributed Artificial Intelligence (DAI) for IoT security, focusing on how local, cooperative intelligence can support intrusion detection, anomaly recognition, secure data processing, and collaborative defence. We synthesise the current literature on Federated Learning (FL), Multi-Agent Systems, and related approaches, highlighting their benefits, limitations, and practical deployment constraints. Particular attention is given to critical infrastructure contexts, where resilience is essential for operational continuity and public safety. The review concludes by outlining key gaps and future research directions for DAI-enabled IoT security.

## 1. Introduction

The Internet of Things (IoT) has become widely deployed across consumer, industrial, and critical infrastructure environments. IoT systems consist of interconnected devices, sensors, and communication technologies that support data collection, automated operation, and context-aware decision-making. These devices typically communicate through wireless and low-power networking standards such as IEEE 802.15.4 [1], NFC, 6LoWPAN, MQTT, and Bluetooth Low Energy [2,3,4,5,6].

In this context, Distributed Artificial Intelligence (DAI) is attracting growing attention. Unlike classic, centralised systems, DAI is based on the cooperation of many entities—devices, computers, or users—who make decisions locally but work together to achieve a common goal. This model enables faster responses, avoiding network congestion and limiting data transfer to central servers [7,8,9].

DAI offers specific benefits. It facilitates automation, speeds up system response times, and enhances user privacy because data does not need to be constantly transmitted elsewhere. In addition, local decision-making allows devices to continue operating effectively even when connectivity to a central server is unavailable [10,11,12,13,14].

Organisations increasingly integrate IoT deployments into critical infrastructure, including smart energy grids, industrial control systems, healthcare networks, and transportation systems. These are critical systems because failures or cyberattacks can disrupt essential services, compromise safety, and cause substantial operational and economic damage. The protection of distributed and complex IoT systems has therefore become a fundamental challenge for infrastructure defence. In this context, DAI methods are particularly relevant because they support more resilient and responsive protection strategies in distributed environments [15].

Unlike many existing reviews that focus primarily on attack taxonomies, learning paradigms, or deployment settings, this review examines DAI in relation to the specific security functions it supports in IoT environments. To do so, it introduces a function-oriented taxonomy that organises DAI approaches by security function, threat context, and deployment constraints. This framework enables a clearer comparison of how distributed intelligence contributes to intrusion detection, anomaly recognition, secure data processing, and collaborative defence, while also distinguishing security effectiveness from deployment feasibility in resource-constrained IoT settings.

The popularity of security systems using ML and deep neural networks is also growing. Such systems analyse network traffic, detect unusual behaviour and can react independently. By combining with DAI, they can be launched without relying on central servers, thereby providing greater scalability and resilience to failures [16].

There is also growing interest in edge AI, which involves transferring artificial intelligence directly to edge devices. Edge AI reduces latency, increases privacy and reduces cloud load. Combined with DAI, it provides a solid foundation for creating more secure and autonomous systems, especially in resource-constrained environments [17].

### 1.1. Motivations and Contributions

Existing research on AI for IoT security remains fragmented, with many studies focusing on specific techniques, attack types, or deployment settings rather than on the broader security functions supported by distributed intelligence. This review addresses that gap by providing a structured analysis of DAI-based approaches to IoT security. The main contributions of this study are as follows:

- We propose a function-oriented taxonomy of DAI-based approaches for IoT security, organised around security tasks, threat context, and deployment constraints rather than solely by application domain or learning paradigm.
- We conduct a structured literature review that synthesises how DAI contributes to the development of IoT systems that are more autonomous, decentralised, and responsive to changing operational conditions.
- We examine key distributed techniques, including Multi-Agent Systems, Federated Learning, and distributed deep learning, and assess their roles in IoT security, with attention to both their advantages and limitations.
- We identify major research gaps and technical constraints and outline future directions, including the integration of DAI with edge computing and trustworthy AI frameworks.
- We provide a practical reference for researchers and practitioners interested in designing more secure, intelligent, and scalable IoT solutions supported by DAI.

### 1.2. Methodology

This review adopts a structured review approach to examine the role of DAI in IoT security. It focuses on DAI-based approaches that directly support IoT security functions, including intrusion detection, anomaly detection, secure data processing, and coordinated response mechanisms. Studies were included when they made a substantive contribution to the intersection of DAI, IoT, and security, either by proposing a DAI-based security method, evaluating its performance in an IoT-related context, or discussing implementation challenges and practical deployment constraints. Works were excluded when they addressed AI or IoT security only in general terms without a clear connection to distributed intelligence, or when they did not provide sufficient technical or analytical detail to support the thematic synthesis.

To complement the qualitative synthesis, each included study was coded using a predefined set of variables, including publication year, application domain, security function, dataset or evaluation setting, method category, and reported deployment constraints. The statistical figures and coverage tables were then generated by aggregating frequencies across these coded entries. The quantitative component is descriptive and comparative in nature; it is not intended as a meta-analysis or as an effect-size estimation procedure.

Compared with prior reviews, this study differs in both scope and analytical depth. Many existing surveys organise the literature around individual methods, application domains, or sequential, study-by-study descriptions, whereas this review groups the evidence by security function, threat context, and deployment constraints. This allows a more systematic comparison of DAI approaches in IoT security and makes it easier to identify where the present review extends beyond earlier work. Differences in reported outcomes across the literature are largely attributable to variations in datasets, threat models, deployment assumptions, evaluation metrics, and device-level constraints, which makes direct comparison difficult unless the studies are interpreted within a common analytical framework.

Rather than performing a quantitative meta-analysis, the paper provides a qualitative synthesis of the literature to identify key trends, practical challenges, and open research directions. This approach is intended to provide a clear, focused overview of the current state of research on DAI-enabled IoT security.

To better frame the scope of this review, it is important to distinguish between the main distributed intelligence paradigms considered in the literature. Distributed Artificial Intelligence (DAI) serves as an umbrella term for intelligent processing and decision-making performed across multiple nodes or entities. Federated Learning (FL) is a distributed learning paradigm that enables local model training while keeping raw data on-device. Multi-Agent Systems (MAS) focus on coordinating autonomous agents that interact and cooperate to achieve shared goals. Distributed deep learning (DDL) refers to the distributed training or inference of deep neural networks across multiple computing resources. Although these approaches are related, they address different security problems and operate under different assumptions.

In the context of IoT security, DAI can improve local response, scalability, privacy preservation, and resilience against single points of failure. However, it does not eliminate fundamental security risks such as compromised nodes, poisoning attacks, adversarial manipulation, insecure communication channels, or resource exhaustion. For this reason, our review interprets DAI in terms of explicit threat contexts and deployment constraints, rather than treating it as a universal solution to all IoT security challenges.

To complement the qualitative synthesis, this review includes a quantitative overview of the literature through statistical analysis, trend figures, method coverage figures, and structured comparison tables. These elements provide additional evidence on publication patterns, methodological distribution, and coverage across the reviewed literature, thereby strengthening the empirical basis of the review’s conclusions.

### 1.3. Organization

The rest of the paper is designed to present, transparently, an exploration of DAI in the context of IoT security. Section 2 describes the current research on the impact of DAI on the security of IoT networks. Key points and open aspects for further exploration are emphasised. In Section 3, very elementary details of DAI methods are explained. Strengths have been well-documented, particularly in complex, distributed systems such as IoT networks. Section 4 reviews some security challenges IoT systems face, including data breaches, unauthorised access, and system vulnerabilities. The existing challenges necessitate implementing advanced security methods that adapt to diverse situations. Section 5 examines particular implementations of DAI in IoT security systems. The paper analyses systems for intrusion, anomaly, and attack detection, secure data handling, and other applications, assessing their operational success and real-world use. Section 6 will analyse the results by critically evaluating DAI-based solutions and identifying research directions to address current limitations. The final section of this paper presents the main findings while discussing the implications of DAI for IoT security applications. The paper will present potential research directions for this field of study.

## 2. Overview of Existing Studies on DAI Implications on IoT Security

Research on IoT security and artificial intelligence has focused on various aspects over the past few years, with attention to specific methods and applications. The literature lacks comprehensive research on the overall security benefits distributed intelligence provides to IoT systems.

Research on IoT security and artificial intelligence has expanded significantly in recent years, but the literature remains uneven in its treatment of distributed intelligence in security applications. Existing reviews can be broadly grouped into three streams: studies on AI- and deep-learning-based IoT security, studies focused specifically on FL, and studies examining hybrid approaches that combine distributed learning with complementary technologies such as blockchain or advanced networking frameworks.

The first stream includes broader reviews of AI and deep learning for IoT protection. For example, Sarker et al. [18] surveyed intelligent security methods in IoT, while Aldhaheri et al. [8] focused on deep-learning-based intrusion detection and discussed datasets, classification strategies, and resource-related challenges. These studies provide useful technical background, but they mainly emphasise centralised or model-centric approaches and give limited attention to the role of distributed coordination in system-wide IoT security.

Recent studies further show that blockchain-based mechanisms can strengthen trust and collaboration in IoT security, for example, by supporting reputation-driven cross-chain data sharing and lightweight secure collaborative computing in resource-constrained networks [19,20]. These works illustrate that distributed security in IoT is not limited to detection tasks, but also includes trusted coordination, data exchange, and access control in decentralised environments.

A second stream focuses more directly on FL as a privacy-preserving and scalable approach for distributed IoT environments. Venkatasubramanian et al. [21] discussed FL for IoT malware detection, highlighting communication efficiency, privacy preservation, and scalability, while Yaacoub et al. [22] reviewed federated algorithms and defence protocols for dynamic IoT networks. Compared with broader AI surveys, these studies engage more directly with decentralised learning, but they remain primarily centred on FL as a single paradigm rather than on DAI as a broader security framework.

A third stream examines hybrid and supporting technologies that strengthen distributed IoT protection. Rahman et al. [23] explored the integration of FL with Information-Centric Networking, whereas Issa et al. [24] and Ali et al. [25] analysed blockchain-assisted FL and distributed intrusion detection in IoT and industrial IoT settings. These studies extend the discussion toward trust, integrity, and coordination in distributed environments, but they are typically organised around specific enabling technologies rather than around the security functions that distributed intelligence supports.

Taken together, the existing literature confirms a strong interest in decentralised and privacy-aware security mechanisms for IoT. However, prior reviews are usually organised around individual techniques, attack categories, or supporting technologies, which makes it difficult to compare how different DAI paradigms contribute to concrete security functions across deployment settings. This gap motivates the present review, which adopts a function-oriented perspective to relate DAI methods to threat context, practical constraints, and operational security roles in IoT environments.

Taken together, the reviewed studies highlight a growing interest in applying advanced AI methods to IoT security. However, most of them focus either on centralised learning models or on specific technological components, such as blockchain or DL, often without situating them within the broader context of distributed intelligence. While FL is frequently discussed, comprehensive analyses linking DAI concepts to practical, system-wide IoT protection remain limited. This confirms the need for a structured overview that bridges these gaps and clearly illustrates how distributed AI methods are being used—or could be used—to enhance the security of IoT environments. Differences in reported outcomes across the literature are largely attributable to variations in datasets, threat models, deployment assumptions, evaluation metrics, and device-level constraints, which makes direct comparison difficult unless the studies are interpreted within a common analytical framework.

Accordingly, the present review adopts a comparative perspective that examines DAI-based approaches in terms of security function, threat context, deployment setting, and practical constraints. This framing makes it possible to identify recurring design patterns, methodological trade-offs, and differences in maturity across the literature, rather than treating individual studies as isolated contributions.

Compared with prior reviews, the present study differs in both scope and analytical depth. Many existing surveys organise the literature around individual methods, application domains, or sequential study-by-study descriptions, whereas this review groups the evidence according to security function, threat context, and deployment constraints. This allows a more systematic comparison of DAI approaches in IoT security and makes it easier to identify where the present review extends beyond earlier work. In particular, the proposed framework is designed to highlight not only what methods exist, but also how they differ in practical relevance, maturity, and suitability for real IoT environments.

## 3. Distributed Artificial Intelligence Methods

Distributed Artificial Intelligence (DAI) refers to intelligent processing and decision-making distributed across multiple nodes or entities rather than concentrated in a single centralised system. This paradigm has become increasingly relevant in IoT environments, where large numbers of devices generate data in distributed settings and where low latency, scalability, resilience, and privacy preservation are important operational requirements. In this review, DAI is treated as an umbrella concept that includes multiple forms of decentralised intelligence, while still distinguishing among their underlying assumptions and roles.

Within this broader framework, FL is understood as a distributed learning paradigm in which models are trained locally while raw data remain on-device. MAS focus on coordination among autonomous agents that exchange information and act toward shared goals, whereas distributed deep learning (DDL) refers to the distributed training or inference of deep neural networks across multiple computing resources. By contrast, Edge AI is considered primarily a deployment context, and blockchain-based security or swarm intelligence are treated as complementary mechanisms that may support trust, coordination, or resilience in distributed IoT systems. Together, these approaches illustrate the diversity of methods that fall within, or interact closely with, the broader DAI landscape considered in this review.

Several recent studies have explored different aspects of DAI. Janbi et al. [26] provided a detailed taxonomy of DAI systems, covering AI workflows, distribution strategies, infrastructure layers, and applications. They also introduced the Imtidad framework for providing DAI-as-a-service (DAIaaS) across cloud, fog, and edge platforms.

Mwase et al. [27] discussed the challenges of applying AI in resource-constrained settings, such as edge computing. Their work highlights the need to optimise communication between components and to design systems that function well even with limited processing power.

Aminizadeh et al. [28] examined distributed AI applications for medical data analysis through cloud, fog and IoT platforms in healthcare settings. The authors studied how deep learning convolutional networks operate in decentralised systems to securely and efficiently handle health information.

Tanghatari et al. [29] developed a method for training deep neural networks across edge devices that protects data privacy while preserving accuracy. The system utilises knowledge distillation to transfer knowledge from lightweight edge models to a larger, cloud-based model.

Wang et al. [30] developed software-defined mobile edge computing (SD-MEC) through their distributed learning algorithm for IoT computation offloading. The system achieves an equilibrium among energy consumption, latency, and performance requirements, all of which are essential for distributed computing environments.

The review by Rosendo et al. [31] provided an extensive overview of DAI across the edge-to-cloud spectrum. The authors examined well-known machine learning libraries and evaluated existing research obstacles, including standardisation and interoperability issues.

Ben-Nun et al. [32] studied the process of parallelising deep learning models within distributed computing systems. The authors studied concurrency methods, communication approaches, and the performance of asynchronous optimisation techniques.

Other studies applied DAI techniques to specific tasks. Saied et al. [33] reviewed AI-based intrusion detection in IoT networks, categorising approaches based on the learning algorithms used. Kubanek et al. [34] and Kulawik et al. [35] applied deep learning to transportation and infrastructure safety, demonstrating how distributed methods can support real-time decision-making across diverse fields.

**Figure 1:**
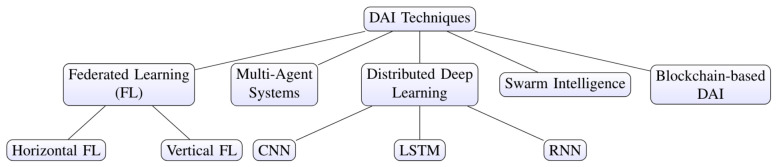
Taxonomy of key DAI methods used in securing IoT systems.

Figure 1 summarises the principal categories of DAI methods discussed in the literature, including FL, agent-based systems, and distributed deep learning. These categories illustrate the diversity of distributed intelligence approaches relevant to IoT security across different application and deployment settings.

To clarify the conceptual scope of this review, it is important to distinguish between related but non-identical terms that appear throughout the literature. DAI is used here as an umbrella concept for intelligent processing and decision-making distributed across multiple nodes or entities. FL and DDL are data- and model-oriented paradigms that distribute training or inference across devices or computing nodes. MAS emphasise autonomous entities that coordinate actions and exchange information to achieve shared goals. By contrast, Edge AI refers to the deployment context in which intelligence is brought closer to the data source. In contrast, blockchain-based security and swarm intelligence are complementary mechanisms that may support trust, coordination, or resilience within distributed systems. In this review, these concepts are analysed together only where they directly contribute to IoT security functions supported by DAI.

## 4. Internet of Things Security

IoT systems consist of interconnected devices, sensors, software, and communication technologies that support data exchange, automated operation, and distributed decision-making across diverse application domains [36,37,38,39]. Their scale, heterogeneity, and continuous connectivity create significant security challenges, particularly with respect to confidentiality, integrity, availability, interoperability, and secure data management [40,41].

From a security perspective, IoT environments are exposed to a broad range of threats, including authentication attacks, communication-based attacks, device compromise, data manipulation, and denial-of-service conditions [42,43,44,45,46,47,48,49,50,51,52,53,54]. These risks are especially important in critical and resource-constrained settings, where delayed detection, weak coordination, or reliance on centralised control can reduce resilience. In this context, DAI-based approaches are relevant because they can support local decision-making, collaborative threat detection, and more adaptive security management. Table 1 summarises representative attack types and common defensive measures.

Despite its potential, DAI in IoT environments also faces several implementation challenges. Most IoT devices have limited computational capacity, making it difficult to run complex models locally. One approach is to combine DAI with edge computing or use lightweight AI architectures. Another challenge is the lack of standardised security protocols across platforms, which complicates integration. The ongoing challenges of false positives and adversarial attacks persist in the field. The combination of continuous training with proper model design techniques helps minimise these security risks.

Implementing DAI at scale faces several operational challenges. The deployment of DAI at scale requires significant investments in high-performance computing systems, reliable storage solutions, and improvements to network infrastructure. The deployment of qualified personnel, together with compliance efforts under the General Data Protection Regulation (GDPR) [69], is essential. The implementation of scalability must strike a balance with performance requirements, privacy needs, and efficiency standards.

The importance of AI and DAI in protecting IoT ecosystems continues to grow. The evolution of these technologies provides robust security tools for threat detection, response, and prediction, but requires careful implementation that accounts for technical limitations and operational and regulatory constraints.

**Figure 2:**
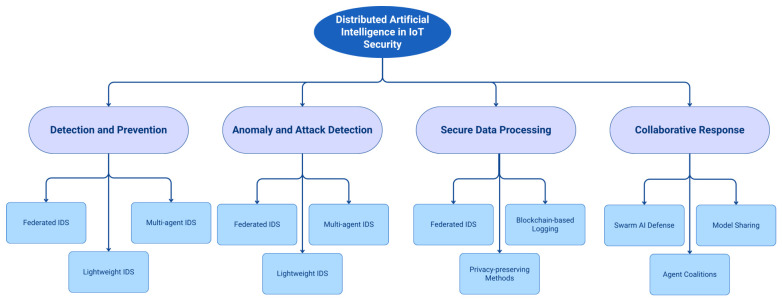
Conceptual classification of the main DAI-based security functions identified in the reviewed literature. The figure groups recurring applications into intrusion detection and prevention, anomaly and attack detection, secure data processing, and collaborative response mechanisms.

To synthesise the reviewed literature, the main DAI applications in IoT security can be organised into a small set of recurring security functions. This classification was derived from repeated patterns identified across the surveyed studies. Figure 2 summarises these functions and their role in structuring the review.

The classification highlights how DAI supports IoT security not through a single mechanism, but through multiple function-specific roles that operate under different threat conditions and deployment constraints.

## 5. Distributed Artificial Intelligence Implications on IoT Security

Communication security between IoT devices requires special attention from network administrators and service designers, as these devices are vulnerable to hacking. From an IoT security perspective, we highlight three crucial areas where we can employ DAI methods. The first is the so-called intrusion detection system (IDS) or intrusion prevention system (IPS), which monitors networks or systems for malicious activity. Next, we can apply DAI methods to specific cyberattacks, such as (D)DoS and malware, which can be particularly dangerous for IoT devices. The third aspect is secure data processing between IoT devices. We can also identify other solutions to improve security in IoT environments.

### 5.1. Intrusion Detection Systems

Javadpour et al. in [70] focused on the Cloud Internet of Things environments. They noticed that these environments are vulnerable to cyber threats, making intrusion detection and prevention systems essential for securing them. However, existing methods have limitations, such as the inability to detect unknown attacks and vulnerability to a single point of failure. Javadpour et al. proposed a novel distributed multi-agent IDPS (DMAIDPS) that overcomes these limitations. The system uses multiple agents to learn network behaviour and performs a six-step detection process. When an intrusion is detected, a prevention server configures firewall rules to block the attack. The proposed system is flexible, allowing for future extensions without affecting its structure. The detection procedure consists of six sequential steps: data gathering, pre-processing, clustering, identifying similar data, generating association rules, and decision-making. The authors used the K-means and K-nearest neighbour algorithms. The system’s performance was evaluated using two commonly used datasets and compared with other detection methods, demonstrating improvements in recall, accuracy, and F-score.

Arkan et al. [71] focused on WSN. They are crucial for the Internet of Things, but they face challenges such as high power consumption, limited flexibility, and programming difficulties. Also, software-defined wireless sensor networks (WSNs) integrate WSNs with software-defined networks to improve security. The authors proposed a novel architecture with an unsupervised intrusion detection algorithm that utilises a hierarchical approach to enhance the security of an integrated software-defined WSN. The authors noted that sensors are not entirely dependent on the software-defined wireless sensor network controller but instead run the appropriate intrusion detection algorithm module locally at the layer. Data analysis results are sent to the software-defined wireless sensor network controller, and decisions are made after checking data normality or abnormality. The proposed architecture detects abnormal traffic up to 97%, improving the speed and accuracy of attack detection at the controller plane.

In [72], Idrissi et al. considered the impact of centralised ML-based anomaly detection methods on improving network intrusion detection systems. Given that FL has emerged as a solution that allows distributed clients to train a shared model while preserving local data privacy, the proposed Fed-ANIDS is a novel intrusion detection method. This autoencoder-based distributed network intrusion detection method leverages anomaly detection and FL to address these issues. The method achieved high performance in various metrics while preserving data privacy. The FL framework (FedProx) outperforms other Generative Adversarial Network (GAN)-based models, achieving higher detection accuracy and fewer false alarms.

Li et al. in [73] addressed the need for architecture-specific intrusion detection systems in IoT environments by proposing a cooperative intrusion detection system based on software-defined networking (SDN). The method divides the network into subnets, each monitored by a controller node, and uses Black Hole Optimisation (BHO) [74] for feature selection together with a Parallel Convolutional Neural Network (PCNN) for traffic classification. The proposed cooperative strategy also applies majority voting among controller nodes to improve attack detection. The results show that the method achieved accuracies of 99.89% on NSL-KDD and 97.72% on NSW-NB15, indicating improved performance over prior approaches.

In [75], de Oliveira et al. proposed F-NIDS, a distributed intrusion detection system, to overcome privacy limitations in distributed IoT scenarios. It offered high accuracy in data traffic classification, a decentralised architecture for rapid scaling, and greater confidentiality of training data and models. F-NIDS uses FL and Differential Privacy techniques [76] for model protection and is evaluated against three different approaches, demonstrating its ability to predict and determine the nature of the attack. This research revealed that IoT faces challenges in decentralised and scalable intrusion detection. F-NIDS received accuracy, precision, and recall metrics similar to those of traditional centralised learning strategies. The authors recommend this system for privacy-critical environments, such as medical and financial domains, as it enhances data privacy while maintaining acceptable classification performance.

Abid et al. in [77] focused on the security of IoT solutions for Industry 4.0, the new generation of connected and intelligent factories driven by technologies such as artificial intelligence, cloud computing, Big Data, and industrial control systems. The authors noted that these systems pose security challenges due to the extensive daily data generated. They proposed a new distributed intrusion detection approach to enhance industrial control systems by leveraging artificial intelligence methods, Big Data techniques, and a cloud environment. The approach utilised convolutional neural networks (CNNs) to classify industrial data, such as water treatment, and the ITrust platform to collect relevant and representative data. The approach offered several improvements to the field of water treatment, including relevant and representative data, efficient data storage and management, enhanced data quality, the application of CNNs to non-image domains, and improved classification performance. This solution addressed the data collection, storage, pre-processing, transformation, and classification stages, advancing the field and improving the efficiency and accuracy of water treatment processes.

Alevizos et al. in [78] proposed a Blockchain-based Collaborative Distributed Intrusion Detection System for Vehicle Sharing Networks, ensuring data integrity and immutability among vehicles and roadside units. The system uses a permissioned blockchain with an allowlist of cryptographic hashes of benign social applications. The system calculates the cryptographic hash of new or modified applications and commits it to the blockchain for whitelisting. The paper also introduced a novel ledger-query strategy that, with modifications, can be applied to large-scale VSNs. The proposed solution addresses performance limitations in large-scale application contexts. The evaluation findings demonstrated a substantial improvement in transaction processing capacity, reaching a maximum of 1991 transactions per second. The CPU and memory performance of all peers declined, suggesting improved resource management. The comparative chart of overall time-to-completion clearly showed that the DTS outperformed the default query technique in speed.

Khonde et al. in [79] proposed a blockchain-based framework for a distributed IDS that enables the transfer of novel signatures between nodes. Attack detection was based on anomaly detection, and a signature was created, validated, and deployed to the network. This secure architecture provided fault tolerance and a single source of truth, ensuring data retrieval even in the event of node failure. It is unique because only trusted nodes can transmit the signature, making it a top solution for distributed IDS networks. The authors evaluated blockchain performance and found that Hyperledger Fabric v2.0 [80] outperforms v1.0 in execution time, latency, throughput, and transaction processing time. A proposed blockchain-based IDS improved signature detection performance by 2%, detection rate by 3%, and false alarm rate by 1.7%. However, bandwidth overhead and processing time may limit its use.

Alferaidi et al. in [81] proposed a distributed combined DL intrusion detection method for the Internet of Vehicles, utilising the Apache Spark v2.3.0 paired with Java (JDK) 1.8 and Scala 2.11.8. framework [82]. They employed a combination of CNN and Long Short-Term Memory (LSTM) methods. Comparative experiments revealed that due to the slow detection speed and low detection efficiency of big data in IDS, the advantages of distributed frameworks and DL algorithms are fully leveraged, and a distributed architecture is combined with the DL CNN-LSTM algorithm. After pre-processing and other methods, data standardisation improves detection efficiency and detection time. Experimental verification on the NSL-KDD [83] and UNSW-NB15 [84] datasets demonstrates that the DL algorithm, specifically CNN-LSTM, utilising the Spark framework, is comparable to other DL algorithms. It reduces training and testing time, improves detection accuracy, and meets the real-time requirements of intrusion detection, thereby satisfying the actual needs of the Internet of Vehicles.

Latif et al. in [85] introduced a compact and highly efficient random neural network for detecting intrusions in an IoT environment. The suggested method was highly suitable for deployment in resource-constrained IoT networks, thanks to its inherent generalisation capabilities and decentralised nature. The proposed model was thoroughly evaluated using the ToN_IoT dataset. The experiments were conducted using several hyperparameters, and the effectiveness of the proposed DnRaNN was evaluated using multiple performance metrics. The conclusions of the suggested study offer recommendations and insights for binary-class and multi-class scenarios. The proposed model achieved attack-detection accuracies of 99.14% and 99.05% for binary and multi-class classification, respectively.

Sun et al. in [86] proposed a new attack detection algorithm utilising a blockchain-based distributed FL model. The model protects data privacy and ensures secure coordination. It used a collaborative model, a parameter verification mechanism, and a proof-of-stake consensus mechanism, encouraging legitimate entities to participate more actively. The algorithm also introduced a novel intrusion detection method based on an end-to-end clustering approach to distinguish among different attack types. Distributed entities can collectively train the global intrusion detection model. The efficacy of the proposed model is validated through comparative experiments conducted on the widely used AWID dataset.

Thein et al. in [87] investigated an FL-based intrusion detection system for IoT data to address challenges such as data heterogeneity and poisoning attacks. The authors proposed a personalised FL-based IDS approach to handle imbalanced data distributions and poisoning attacks. They introduced mini-batch logit adjustment loss to local model training to obtain a personalised model tailored to each local data distribution. A poisoned client detector was designed on the server to identify malicious agents by comparing the cosine similarity of local models to the non-poisoned client’s centroid. The detector identifies poisoned clients and restricts their aggregation on the global intrusion detection model. Compared to baseline methods, the proposed model can detect poisoning attacks without compromising performance. It was evaluated using the N-BaIoT dataset to study its effectiveness. The study examined various data-distribution scenarios and experiments involving different poisoning attacks, comparing the poison detector against other state-of-the-art methods. The results show that the proposed method effectively detects IoT network traffic anomalies and can reduce the attack success rate, thereby mitigating the negative impact of poisoning attacks on the global intrusion detection model.

Jin et al. in [88] proposed a novel IDS framework, FL-IIDS, to address catastrophic forgetting in FL. The framework employs a novel loss function for local model training, assigning different learning weights to new and old classes to reduce the learning rate of new classes and enhance the memory of old classes. The sample label smoothing loss function utilises knowledge distillation to enhance the local model’s memory for specific old classes. The relay client fusing sample reconstruction mitigates catastrophic forgetting globally without compromising data privacy. Extensive experimental results demonstrate that the proposed framework enhances memory capability for old classes without compromising detection effectiveness for new classes. The framework also incorporates recall-based and regularisation-based approaches to mitigate local-side catastrophic forgetting.

Khan et al. in [89] attempted to integrate IoT and medical IoT networks using an FL model to monitor and analyse medical data in decentralised environments, thereby preserving users’ privacy. The research proposed a privacy-preserving FL-based IDS model, Fed-Inforce-Fusion, to identify cyberattacks in distributed IoT-based medical systems. The model utilised Reinforcement Learning (RL) to learn the latent relationships within medical data, with a focus on addressing security and privacy issues. The Fed-Inforce-Fusion system enabled distributed system health nodes to train a comprehensive IDS model in a privacy-preserving manner collaboratively. A fusion strategy was employed to enhance detection performance and minimise communication overhead. The proposed framework was validated against compelling benchmark approaches. The results confirmed that the devised method is robust and reliable in detecting sophisticated attack vectors, outperforming benchmark methods with 99.40% accuracy and 98.99% detection rate.

Wang et al. in [90] employed FL for anomaly detection in IoT solutions. They discovered that FL models enable the faster design of shared, ML-based attack-detection systems while preserving local data privacy. In their method, each client can download a local model and train it on local data, with the updated parameters uploaded to the global model. The global model will receive samples from each client and aggregate them to improve its performance. This single federated process round is repeated for all clients to achieve reliable performance. They based the local model for anomaly prediction on mutual information-based feature selection and deep neural network-based prediction. The demonstration results for the proposed federated model show superior performance compared to existing federated DL methods for anomaly detection in IoT. The federated technique achieves superior training speed compared to the centralised strategy. The model’s accuracy and robustness are assessed using classification and regression metrics. The suggested FMI-DNN achieved an accuracy of 99.4% and an error rate of 0.142. The suggested model had an ROC value of 0.9, demonstrating its effectiveness in detecting anomalies.

Friha et al. in [91] introduced FELIDS, an FL-based intrusion detection system designed to enhance the security of agricultural IoT infrastructures. The FELIDS system ensured data privacy by employing local learning, in which devices enhanced their knowledge by exchanging model updates with an aggregation server, thereby producing a more accurate detection model. The FELIDS solution utilised three DL classifiers—deep neural networks, convolutional neural networks, and recurrent neural networks—to mitigate attacks against agricultural IoTs. The authors analysed the effectiveness of the proposed IDS using three distinct data sources: CSE-CIC-IDS2018, MQTTset, and InSDN. The findings indicated that the FELIDS system surpasses traditional, centralised ML (non-FL) approaches in safeguarding the confidentiality of IoT device data and achieves the highest precision in threat identification.

### 5.2. Specific Cyberattack Detection

Alohali et al. in [92] focused on deficiencies in achieving security against specific attacks, such as jamming [93], denial of sleep [94], manipulation [95], and cheating, in IEEE 802.15.4-based WSNs. The swarm intelligence (SI) algorithm [96] can adapt to changes in network structure and traffic. The advanced ants in the SI technique communicate with each node by either sending messages to a single recipient (unicast) or broadcasting messages to multiple recipients (broadcast), depending on whether the channel information is available at the end of the channel. Assuming the channel information is available, the ants select the next hop at random. Data from the retrograde antenna confirm the intruder’s presence over an extended period and prevent the use of a specific communication channel, thereby enhancing the efficiency of the wireless sensor network. The proposed technique achieves a packet delivery rate of 0.4441 and 0.6633. The effective packet delivery ratio demonstrates the efficacy of the proposed approach. The simulation results demonstrate the efficacy of the suggested swarm-based defence technique in terms of attack rate, duration, and the number of attackers, compared with existing strategies. However, it should be noted that the study is based on simulation, so its practical deployment readiness in real IEEE 802.15.4-based WSNs still requires further validation [92].

Bhandari et al. in [97] focused on challenges in Smart Environments within IoT infrastructures. They proposed an AI-enabled approach to detect malware attacks on IoT devices in Smart Environments. The approach used a multi-agent network of AI models, with the most complex models trained in the cloud environment and others in Fog/Dew. The approach detects attacks and malware on IoT devices, facilitates live network traffic tracking, identifies security issues, reduces maintenance effort, and performs performance and concurrency testing. The study suggests that this method is feasible for efficient implementation in real-world, in-production Smart Environments. The authors implemented the proposed model on two specific IoT gateways. They witnessed highly encouraging outcomes. On average, network bandwidth increased by less than 30 kb/s, while CPU usage rose by only 2%. Also, NVIDIA Jetson and Raspberry Pi devices utilise 0.42 GB and 0.2 GB of physical memory, respectively. The authors summarised that their framework accurately and efficiently identifies malware and assaults in Smart Environments.

Alamer et al. [98] noted that the rise in malware attacks has necessitated the development of advanced malware detection methods. Due to limited dataset availability, current detection systems, such as ML-based ones, often fail to detect new or unknown malware. Additionally, they observed that the Internet of Things has led to an increase in malware types, rendering current detection systems outdated. The authors proposed PPFL-SC, an efficient privacy-preserving FL with secure collaborative verification. They used group-oblivious signcryption for FL and the Stackelberg incentive model to encourage IoT devices to collaborate in a malware-detection model. The security analysis confirmed that PPFL-SC fulfils all security requirements for privacy-preserving FL, and experiments on real-world datasets validate its efficiency and practicality.

Kumar et al. in [99] aimed to address security challenges in blockchain-enabled IoT networks, particularly in the mining pool. They proposed a distributed IDS based on AI-enabled fog computing, which integrates mining pools to detect Distributed DoS attacks (DDoS). This method will be part of the framework for deploying anomaly-based IDS in a blockchain IoT environment. The research used AI as an analytical tool, decentralising cloud-based centralised security mechanisms, and fog computing to handle data analysis and security concerns at the edge of networks. The proposed detection system was evaluated using ML algorithms, an IoT-based BoT-IoT dataset, and various evaluation metrics. The experimental results demonstrated high accuracy in both binary and multi-class classifications. The training period for binary classification was shorter than for multi-class classification. This suggests that ML approaches have significant potential to revolutionise cybersecurity by improving threat detection in distributed blockchain-IoT/Fog environments.

Myneni et al. [100] observed that the increasing number of IoT edge devices has led to a rise in DDoS attacks, and current defence mechanisms are insufficient due to high false-positive rates. They proposed SmartDefense, a distributed DDoS detection and mitigation framework based on edge computing. The system utilised a Feed-Forward Neural Network (FFNN) at the consumer edge and an LSTM at the edge of the Internet service providers. The framework blocks easy-to-detect DDoS attacks close to the source and identifies and blocks attacks by correlating information across multiple customer edge networks. It reduces the unnecessary bandwidth that DDoS traffic consumes on residential edge networks, routing it to ISP edge networks. SmartDefense improved detection and mitigation rates, catching over 90% of DDoS traffic at the source and 97.5% at the provider’s edge.

Jmal et al. in [101] proposed a holistic security approach for software-defined IoT infrastructure with multiple controllers. It used blockchain and an artificial neural network to maintain a secure record of analysed and malicious traffic, preventing future attacks. This approach addressed the centralisation issue of software-defined networking and enhanced the performance of IoT networks. The study explored DDoS attacks in IoT and SDN and highlighted their mitigation capabilities. They concluded that it is essential to consider security, cost, and performance trade-offs to address IoT system challenges and to evaluate requirements and constraints. Additionally, it is necessary to conduct risk assessments, adopt new architectures, and utilise adaptation techniques for blockchain and SDN controllers.

Amiri-Zarandi et al. in [102] presented a GAN-based model poisoning attack [103] to demonstrate the vulnerability of FL in uncertain environments. The attack demonstrated the benefits of a trust-based collaborative learning process, preventing adversaries from compromising the final model. The study also proposed a trust-based federated approach to intrusion detection in Social IoT, enabling cooperative, privacy-preserving intrusion detection while preventing malicious actions by untrusted participants. A collaborative feature selection method was also designed to accelerate learning by leveraging cooperation from trusted participants to select valuable features. A collaborative feature selection method should enhance performance and reduce system overload, improving overall efficiency. The authors summarised that the proposed model improves accuracy and F-score compared to self-learning and federated approaches, reducing disruptions. It outperforms independent IoT feature selection. Future work aims to utilise explainable AI techniques to enhance the proposed IDS, elucidate malicious behaviour patterns, and enhance security practices.

Julian et al. in [104] deployed a distributed framework utilising DL to mitigate several sources of vulnerability within a unified defence solution. The evaluation involved two distinct DL models: an FFNN and an LSTM model—the proposed distributed attack detection architecture aimed to enhance intrusion detection capabilities in IoT Fog computing architectures. The architecture consisted of three layers: Edge, Fog, and Cloud, which contained a global model updated by fog nodes, trained the DL models, and provided pre-processed raw data to fog nodes. This architecture improved network performance, reduced latency, and enabled rapid response to critical actions. The models were assessed using two distinct datasets to measure their performance and ability to detect various types of assaults. The results indicated that the proposed distributed system is highly efficient in identifying various cyber-attack types, achieving up to 99.95% accuracy across different configurations.

Doriguzzi-Corin et al. in [105] proposed an Adaptive FL Approach to DDoS attack detection (FLAD), a solution for cybersecurity applications that uses an adaptive mechanism to orchestrate the FL process by dynamically assigning more computation to members whose attack profiles are more challenging to learn. Using a recent dataset of DDoS attacks, FLAD outperforms state-of-the-art FL algorithms in terms of convergence time and accuracy across a range of unbalanced datasets comprising heterogeneous DDoS attacks. The approach was robust in a realistic scenario in which the DL model was retrained multiple times to introduce new attack profiles into a pre-trained model. The high-level idea behind FLAD is to involve only clients whose local validation sets do not yield sufficiently good results with the current global model. For such clients, the amount of computation is determined by the relative accuracy of their validation sets, ensuring that no sensitive data is exchanged between the server and the client. FLAD introduces negligible traffic overhead between clients and servers without disclosing clients’ sensitive data, even during testing.

### 5.3. Secure Data Processing

In [106], Huang et al. investigated the collaborative service caching problem in edge computing from the perspective of IoT service providers. His solution aimed to maximise long-term system utility: the weighted sum of service cost and service latency reduction. The problem was formulated as a multi-agent, combinational, multi-armed bandit problem [107], where edge servers serve as agents, service items are arms, service caching decisions are actions, and system utility represents the reward. A utility-aware collaborative service caching scheme was designed based on multi-agent RN. They conducted extensive experiments to evaluate the performance of the proposed scheme and compare it with four baseline algorithms. The results show that the proposed scheme outperformed the four baseline algorithms.

GabAllah et al. in [108] proposed a multi-tier architecture to distribute the ML task across three tiers. This involves making predictions on individual devices at the edge using locally generated, decentralised data. Conversely, broader projections at a larger scale are assigned to higher tiers, specifically the fog and cloud tiers, using data collected from various edge and fog devices. This is accomplished by selectively broadcasting a subset of the “data of interest” to facilitate the execution of higher-level activities, rather than transmitting the complete raw data over the network. This method enables them to significantly decrease the volume of data sent over the network, circumvent the delays in response time for time-sensitive decision-making, and simultaneously retain the advantages of a centralised cloud system that can perform extensive, long-term analysis on a condensed, pre-processed collection of data points that are most pertinent to optimising the model.

Hamdan et al. in [109] employed FL as a developing technique that enables large-scale model learning without transferring data to a central data centre. They proposed a novel edge-computing architecture for large-scale IoT-based cyber-physical systems, utilising distributed multitask learning over edge-computing networks. This architecture combined ML and population-based search algorithms to optimise the learning process. The architecture supported multiple distributed machine models trained using a combination of algorithms. Simulation experiments with real IoT datasets demonstrated that the proposed learning models achieve high-accuracy results comparable to those of single-machine models while utilising edge computing resources more efficiently. This approach benefits large-scale IoT deployments, as local learning is not feasible due to limited resources and computational constraints.

Srirama et al. in [110] observed that IoT data transmission to and from the cloud frequently incurs latency. To solve this problem, they proposed the CANTO framework. This framework is a distributed processing method that uses the actor model to train neural networks on fog devices in an IoT setting. They extended the Akka framework, as proposed in [111], for neural network training. Also, they use data parallelism [32] to parallelise ML models and a centralised, asynchronous weight-update distribution scheme. CANTO introduced a new message type with parameters such as dataset size, activation function, and learning rate. The framework is containerised and deployed in a Docker Swarm 1.2.9, and its effectiveness is demonstrated through IoT-based forest fire prediction, with a focus on accuracy, latency, and load distribution. Also, the framework supports a basic set of functions, including Stochastic Gradient Descent, square loss, ReLU, sigmoid, and softmax.

Ivoghalian et al. in [112] noticed that advanced wireless communication technologies enable large-scale, geographically distributed systems with thousands of devices with conflicting application requirements. These devices must adapt to varying environmental conditions to ensure overall network performance and service stability. They proposed an application-aware adaptive method that allows devices to dynamically configure communication parameters based on their application requirements and environmental conditions. The algorithm, implemented on the modern LoRaWAN network protocol (version 1.0.4), aims to enhance and maintain network performance while enabling each device to meet its specific application requirements. Simulated experiments show improvements in packet delivery ratio and energy consumption. The proposed solution was implemented using the contemporary LoRaWAN network protocol [113].

Aghapour et al. in [114] proposed a framework for distributing the execution of multiple CNNs for IoT users across available edge servers, minimising system delay and energy consumption. The algorithm considered several cloudlets as computing resources, allowing multiple users to be served simultaneously. Distributing layers of a neural network across cloudlets enabled new approaches, such as the early-exit method, to reduce total resource and power consumption. The distribution of deep-network-layer processing across edge servers near IoT devices has not been investigated. The proposed method may assign different CNN layers to edge servers and allocate resources in a multi-user, multi-resource scenario. It employs a two-phase learning algorithm based on Deep Reinforcement Learning (DRN), an improved Salp Swarm Algorithm [115], and two techniques to enhance DRN and accelerate convergence. The proposed algorithm consisted of two phases: offline and online learning. In the offline phase, the deep neural network was trained using the results, and in the online phase, the network was embedded into the system’s learning policy. The orthogonal array was also proposed as an efficient method for determining the optimal hyperparameter combination for deep neural networks. The IDRL algorithm outperforms the full local, offload, and jointly resource-allocation-and-computation-offloading PSO methods [116] for IoT device tasks and cloudlet states. As the computational load increased, it achieved cost reductions of 95%, 14.5%, and 18.4%, respectively. The algorithm assumes fixed cloudlet availability, but future work could consider dynamically variable cloudlet usage. Offloading decisions and resource allocation were optimised at the beginning of each period.

Wang et al. in [117] proposed Reliable FL with Privacy-Preserving for IoT, aiming to enhance security in smart cities by addressing privacy disclosure attacks and improving model reliability. The approach consisted of a lightweight network to hide private data, a multimodal, fine-grained model optimisation to improve accuracy, and adaptive Differential Privacy mechanisms. A simulation experiment on an image classification dataset validated the framework, demonstrating an average accuracy increase of 1.1% over two prevailing methods and a reduction in convergence time of more than 9%. This approach ensured privacy protection while improving model accuracy. The authors concluded that lightweight networks can be vulnerable to white-box attacks, leading to data leakage.

Cao et al. in [118] proposed a Secure and Robust Framework with Trusted Execution Environments for FL. This novel framework was designed to protect data privacy and enable personalised training of non-independent and identically distributed data on IoT devices. Framework used Trusted Execution Environments [119] to safeguard sensitive model components from leaking. It divides models into two layers: local and global, with the local layer retained in the secure world for separate training and the global layer uploaded to the server side for aggregation. Additionally, they proposed a multi-model robust aggregation method based on the membership-degree approach to enhance the robustness of FL under non-independent and identically distributed data. The framework was developed in a simulation environment and evaluated for its performance and defensive capabilities. The results showed that the framework protects the model from membership inference attacks [120] and Byzantine attacks [121], and reduces the success rate of backdoor attacks [122] by 4–10% compared to other robust aggregation algorithms.

### 5.4. Other Solutions

Zhang et al. in [123] proposed a smart industrial IoT-enabled crowd sensing technology for safety monitoring in coal mines, addressing challenges in neural network training and achieving high accuracy in predicting human body position and pressure values, which is not possible with traditional LSTM methods. Using intelligent Industrial IoT-empowered crowd sensing, they applied their method to analyse interactions among individuals, machines, and the environment. This analysis enabled them to predict human movement and the pressure exerted on the supporting machine in a mine, and to determine whether a person is in a hazardous environment. Additionally, they developed a prediction system called Particle Swarm Optimisation–Elman neural network trajectories, which incorporates both the Particle Swarm Optimisation algorithm [124] and the Elman neural network [125,126]. The Elman neural network was optimised using the Particle Swarm Optimisation algorithm, which minimised the impact of the initial weights and thresholds. Also, the authors developed an ADI-LSTM algorithm to forecast pressure values by considering correlations among neighbouring support machines within the same working area, utilising the spatiotemporal distribution of supports, and generating accurate predictions. Crowdsensing technology enables the sensing and computation of individuals, machinery, and the environment in an intelligent industrial IoT coal mine. This technology offered a solution for safety monitoring by optimising DAI.

Filho et al. in [127] employed federated learning methods for botnet detection in IoT networks. The proposed model relied on locally stored data on each device rather than training on a centralised server, and the server aggregated the local updates into a collaboratively trained model while preserving privacy by avoiding raw data transmission. Botnet attacks were identified through decentralised traffic analysis, and the federated approach was used to improve anomaly detection while keeping data at the network edge. The model achieved up to 98% accuracy in anomaly detection using features such as MAC addresses, IP addresses, and source/destination IP addresses during training. Overall, the results indicate that the proposed FL-based solution outperformed a conventional centralised system in attack detection accuracy and better supports privacy-sensitive IoT environments [127].

Antwi-Boasiako et al. [128] proposed a new privacy-preserving distributed deep learning (DDL) framework that addresses certificate issues and key escrow problems in identity-based public key cryptography-based DDL. Existing schemes are secure based on traditional number-theoretic problems, but are not robust or resistant to post-quantum attacks. To address this, the authors proposed a cryptographic primitive, Certificateless Additive Homomorphic Encryption, and formalised its security notions for specific attacks against certificate schemes. They constructed a concrete scheme based on the learning-with-errors assumption to ensure it is post-quantum-resistant. The proposed Certificateless Additive Homomorphic Encryption preserves dataset privacy in DDL and is secure under the learning-with-errors/Decisional assumption. New partial-sharing algorithms have been introduced to reduce the communication costs of transmitting encrypted gradients. The paper removed the requirement for complex certificate management in traditional Public Key Infrastructure-based DDL and eliminated the inherent key-escrow problem in other solutions. The random oracle model [129] was used to test the security of the encryption scheme, assuming the hardness of learning with error or distributed learning with error. Experiments were provided to evaluate the proposed solution. They achieved 97.17% and 97.12% accuracy in selecting the top gradients and in selecting gradients at random, respectively.

Prokop et al. in [130] noted that FL is gaining popularity in ML for its ability to deploy AI systems and train models on private datasets. Additionally, they found that this solution is not ideal because it can be manipulated or intercepted in transit. The authors proposed a solution to ensure model security by encrypting data using AES [131], DES [132], and RSA [133], and storing a checksum on a blockchain. This allowed recipients to verify the model’s accuracy. Their solution combined FL with a blockchain and checksum. Model encryption minimises model manipulation, while checksums verify incoming models. The solution enhances communication security between FL servers and clients, stores confidential information in blocks accessible only to FL clients, and introduces client-generated checksums that the server verifies before aggregation. The authors tested and compared their solution, achieving 81% attack detection with the checksum mechanism.

Salim et al. in [134] proposed an FL-CTIF framework to secure digital twin-based industrial IoT systems against cyberattacks. A specialised cloud-based security auditor investigated vulnerabilities across the industrial IoT network and its sub-departments. The framework utilised information fusion to merge two existing DDoS-based cyberattack detection datasets, thereby enhancing cyber threat intelligence. The FL-based artificial neural network model was designed to reduce the number of training rounds based on satisfaction levels and to detect the absence of improvement in each attack vector’s average accuracy. The model was evaluated and compared with existing research, demonstrating higher accuracy and lower false-alarm rates across the attack vectors of ARP poisoning, SSL-based attacks, and DNS flood attacks. The FL-CTIF framework is suitable for third-party security auditors who regulate local cyber intelligence and prevent cyberattacks.

Mohammed et al. in [135] focused on security issues in healthcare IoT. They proposed an energy-efficient distributed FL offloading and scheduling approach for healthcare systems in blockchain-based networks. The system consists of three physical paradigms: mobile computing as the thin client, fog nodes as the thick client, and cloud computing as the thick client. The objective is to minimise energy consumption during execution. The study presents a blockchain-enabled healthcare system for healthcare workloads on fog and cloud networks. The FL-enabled schemes train and test the healthcare dataset at different nodes, such as fog and cloud nodes, and share the chronic disease dataset with mobile computing for processing. The study also presents offloading and scheduling schemes that utilise the least energy to handle blockchain security while meeting application quality needs. Energy-efficient, lightweight hashing schemes for blockchain technology based on fog-cloud networks are designed.

Based on the established EC network model, Peng et al. in [136] proposed an adaptive collaborative anti-interference mechanism for IoT edge systems. The algorithms combined full-frequency multiplexing and interference mitigation to improve performance in harsh electromagnetic environments. The paper included the construction of an edge computing system model, an Occupy-Interference Mitigation algorithm, and an anti-interference algorithm based on interference mitigation and inter-cell frequency division for interference avoidance. Extensive simulation results on the Mininet platform [137] demonstrated that the proposed algorithms could achieve a lower failure probability than traditional solutions, meeting the requirements for transmitting power, bandwidth, and signal-to-noise ratio. O-IM is suitable for low-power scenarios, whereas interference mitigation and avoidance are better suited to high-power cases.

Atitallah et al. in [138] proposed FedMicro-IDA, a novel data analytical solution that leverages insights from IoT ecosystems using FL and TL methodologies. The solution consisted of software components that supported distributed learning and met the needs of IoT applications. The authors propose an intelligent microservice-based approach that ensures data privacy and security while providing effective analytics to end users. They broke the IoT application into a linkable collection of microservices running across multiple computing layers, focusing on providing adequate service computing distribution. The authors also validated the approach in a malware detection and classification case study using the public MaleVis dataset. The approach’s performance was evaluated using various metrics and microservice execution times. The proposed architecture utilised Federated Transfer Learning to augment and optimise the learning process using several CNN models. CNN model training uses several virtual instances on the edge to utilise its training data. The revised parameters are transmitted to the cloud-based central server for aggregation and processing. After the global models are created, they are utilised at the edge to deliver the necessary data analytics.

### Summary and Observations

The analysis of state-of-the-art systems demonstrates that DAI is a fundamental element in improving IoT security. The combination of FL, MAS, and blockchain-based solutions in DAI enables real-time protection mechanisms that preserve privacy while providing scalable security for intrusion detection, cyberattack prevention, and secure data processing.

IDS systems that use decentralised learning methods via federated or multi-agent architectures demonstrate superior performance to traditional models when data privacy and threat adaptability are essential. Distributed approaches to malware detection and DDoS mitigation demonstrate strong potential for use in edge- and fog-based architectures, which reduce latency while maintaining detection quality.

Multiple ongoing challenges hamper the current progress. Systems need to address problems such as model poisoning, adversarial manipulation, and performance trade-offs arising from decentralised processing. The evaluation process currently takes place in simulated environments, demonstrating the need for more real-world testing.

The research demonstrates that Distributed Artificial Intelligence offers effective security solutions for complex IoT systems, although it does not provide a comprehensive solution. Future development should focus on creating hybrid systems that integrate DAI with edge AI and explainable AI, along with formal security guarantees, to establish trustworthy systems ready for large-scale deployment.

## 6. Discussion

This section summarises the reviewed papers on DAI solutions for IoT security. Table 2, Table 3, Table 4 and Table 5 compare the main methods and application areas, including IDS, specific attack detection, and secure data processing.

To move beyond a purely descriptive account of the literature, this discussion adopts a comparative perspective that emphasises how the reviewed approaches differ in datasets, evaluation metrics, scalability assumptions, attack resistance, privacy preservation, real-world applicability, and deployment readiness. These dimensions provide a more meaningful basis for interpretation than sequential study summaries alone, because they reveal both the relative maturity of different DAI techniques and the practical trade-offs associated with their use in IoT security.

Across the reviewed studies, approaches based on Federated Learning and multi-agent coordination are generally better suited to privacy-sensitive and distributed environments. In contrast, blockchain-enhanced and edge-deployed solutions tend to prioritise trust, integrity, and low-latency response at the cost of higher communication or computational overhead. Distributed deep learning methods often achieve strong detection performance, but their deployment value depends on whether the underlying infrastructure can support the required processing and coordination demands. These differences show that technical accuracy alone is insufficient to assess a method’s suitability for IoT security.

Accordingly, the comparative value of DAI-based solutions should be judged not only by performance scores but also by how well they balance accuracy, efficiency, privacy, scalability, and operational feasibility under realistic deployment conditions.

To complement the qualitative synthesis, the review incorporates an empirical overview of the literature through structured tables and method-coverage analysis. The tabular presentation of IDS, specific attack detection, secure data processing, and other DAI-enabled solutions makes it possible to observe how frequently particular techniques appear in the literature and how the evidence base is distributed across application areas.

This empirical organisation strengthens the discussion by making methodological concentration, coverage gaps, and the relative prominence of specific DAI techniques more visible. In particular, the reviewed literature places a strong emphasis on Federated Learning-based security solutions. In contrast, other approaches, such as MAS, swarm intelligence, and distributed deep learning, appear less frequently or in more specialised contexts. The resulting distribution indicates that the field is still unevenly developed and that some promising directions remain underexplored.

These empirical patterns support the conclusion that DAI is already effective in several IoT security settings, but the current evidence base remains dominated by a limited set of techniques and evaluation scenarios. This makes additional benchmarking, broader deployment testing, and method comparison essential for future work.

Practical deployment remains a central constraint for DAI-based IoT security. Many of the reviewed approaches reduce reliance on centralised infrastructure, but they also introduce non-negligible computation, communication, latency, and energy costs that must be considered when assessing suitability for resource-constrained IoT nodes. Federated Learning and multi-agent approaches are generally more compatible with edge deployment, but their effectiveness depends on how efficiently model updates, coordination, and local inference can be performed under heterogeneous device capabilities. Distributed deep learning and blockchain-assisted mechanisms may offer stronger functionality or trust guarantees, but they often require higher resource investment and therefore face greater limitations in low-power or bandwidth-constrained environments.

Table 2 summarises the proposed DAI techniques used in IDSs for IoT environments. The most popular DAI technique in the reviewed IDSs was FL. FL dominates because it fits well with privacy-preserving distributed learning. The characteristics of the examined methods depended on DAI techniques and intrusion detection system applications.

Table 3 provides an overview of the proposed DAI techniques used in specific attack detection systems for IoT environments. For the overviewed specific attack detection systems, the most popular DAI techniques were FL and a combination of FFNN and LSTM. Most of the reviewed research focused on detecting DDoS attacks.

Table 4 summarises the proposed DAI techniques used in secure data processing systems for IoT environments. For the overviewed systems, the most popular DAI techniques were FL and a combination of CNN and DRN. The characteristics of the examined methods depended on the systems’ applications. Primarily, these systems focused on ensuring the correct operation of services in distributed IoT environments across various applications. This suggests that DAI is also valuable for reducing latency and preserving privacy in distributed IoT workflows.

Table 5 summarises the proposed DAI techniques used in other security solutions for IoT environments. In these solutions, the most popular DAI technique was FL. FL also appears in non-core security scenarios, which shows its versatility across IoT use cases.

The reviewed evidence confirms that IoT security solutions must account for resource constraints, heterogeneity, and compatibility issues. These factors strongly influence how DAI methods can be deployed and evaluated in practice.

Moreover, because they are connected to the Internet, they are exposed to various attempts to hack the network or intercept traffic, which may, in turn, lead to privacy violations or data leaks. The attacker may also use the hacked IoT device to perform another attack. Creating a reliable detection system is a significant obstacle to ensuring IoT security.

Anomaly detection is valuable in IoT because it can identify unknown or zero-day threats without relying on fixed signatures. The reviewed studies suggest that this capability is especially useful in dynamic and heterogeneous environments.

However, it is worth remembering that no anomaly detection system is perfect, and there is always a risk of false alarms or certain types of attacks going unnoticed. Therefore, it is generally recommended to employ multiple layers of security to enhance the network’s overall resilience.

The reviewed studies show that DDoS is the most frequently addressed specific attack in DAI-based IoT security research. This is unsurprising, given the high impact of DDoS on availability in resource-constrained IoT systems. DAI-based approaches are therefore particularly relevant for distributed detection and mitigation near the source.

Secure data processing is particularly relevant in medical and industrial IoT, where sensitive data and operational continuity are both critical. DAI supports this area by enabling more resilient and privacy-aware distributed processing.

Based on the overview research, we can highlight the following. Mitigating the risk of cyberattacks in IoT through DAI methods involves leveraging decentralised decision-making, detection, and response capabilities across the IoT ecosystem. It is possible to apply some secure strategies. Using edge computing to process data closer to the source will reduce the need to transmit sensitive information to centralised servers. This improves latency and minimises the attack surface for potential cyber threats. The distributed approach helps detect and prevent cyberattacks closer to their sources, enhancing overall network security by deploying firewalls and IDS at various points within the IoT network, including edge devices and gateways.

Additionally, integrating blockchain or distributed ledger technology may secure transactions and data exchanges within the IoT network. Blockchain’s decentralised and tamper-resistant nature can enhance the integrity and security of data. Applying FL techniques for anomaly, attack, or intrusion detection in IoT devices enables them to learn and detect anomalies collaboratively without sharing raw data, thereby enhancing the network’s overall security.

FL is the most promising and most frequently used DAI approach in the reviewed literature. It supports privacy-preserving collaborative learning, reduces communication overhead, and scales well across distributed devices. At the same time, it still requires careful handling of heterogeneity and adversarial risks.

Additionally, FL enables us to personalise the model for each device, accounting for local data differences. Users can utilise more personalised models, resulting in a better user experience. These methods also reduce computational costs. The central server does not need to process all the training data, which can reduce computational costs for storing and processing large volumes of data. This process can be more resource-efficient compared to traditional ML models. Because data remains on local devices, there is less risk of attacks on the central server to access sensitive information. FL enables us to easily integrate new devices into the learning process, making it scalable and adaptable as the number of participants grows. For data regulated by law or considered sensitive, FL allows us to comply with privacy regulations while minimising the risk of breaches.

Addressing these security considerations can make IoT environments more resilient to poisoning attacks and other cybersecurity threats. Integrating these DAI methods can also enhance the security posture of organisations’ IoT deployments and better defend against cyber threats. Adopting a proactive, adaptive security strategy is crucial to keeping pace with evolving threats in the dynamic IoT landscape.

In the discussion of IoT security using DAI solutions, it is worth mentioning another aspect often overlooked in the literature but of great importance: algorithmic transparency, i.e., explainability. Algorithmic transparency remains an important limitation because many reviewed systems report performance gains without explaining individual decisions. This is a major concern in critical applications where trust and accountability matter.

In summary, specific DAI techniques have varying levels of utility depending on the type of threat. Table 6 compares the most common attacks in the IoT environment with the DAI methods that are effective in detecting or mitigating them. While this comparison is illustrative and does not encompass all possible dependencies, it can serve as a practical reference point for further research and implementation.

A consistent comparison between distributed and centralised methods is necessary to determine when decentralisation offers a real advantage. In practice, distributed approaches are most beneficial when privacy preservation, local autonomy, and reduced raw-data transfer are primary requirements, and when the available infrastructure cannot support efficient centralised processing. Under these conditions, Federated Learning and other decentralised paradigms may deliver performance comparable to centralised baselines while offering stronger privacy and greater deployment flexibility. However, this advantage should be assessed using the same data splits, evaluation metrics, and operational assumptions, since apparent gains can otherwise reflect differences in setup rather than genuine methodological superiority.

### 6.1. Example Use Case

Many of the presented solutions are conceptual or technical in nature, but lack a broader contextualization of their applications. Meanwhile, a simple implementation example could help clarify the practical benefits and limitations of DAI systems.

For example, in a hospital environment—where dozens of devices (for example, vital signs sensors, infusion pumps, personnel location systems) interact in real time—the use of DAI can enable faster detection of anomalies (for example, in the work of a ventilator), without the need to send data to the cloud. Local decisions made by agent systems can enable real-time response, increasing patient safety even in the event of a network failure.

Similar solutions could be used in smart cities, for example, to detect irregularities in road traffic or air quality, where decentralisation helps reduce delays and dependence on central infrastructure.

Technology is associated not only with opportunities, but also with specific social and ethical challenges. DAI can support decentralisation, promote cooperation and facilitate access to services. But it can also raise questions—who is responsible for the decisions made by autonomous systems? Are the systems free from bias? Do they exclude anyone?

### 6.2. Limitations and Future Challenges

The increasing adoption of DAI-based methods for IoT system security remains limited by multiple unresolved issues.

The main challenge of distributed intelligence is its high computational requirements. Many IoT devices operate under strict resource constraints—limited memory, processing power, and battery life—which makes it challenging to deploy complex models locally. While techniques such as model pruning or edge offloading help reduce these requirements, the problem is far from solved, especially in large-scale, heterogeneous networks.

Another vital issue is adversarial robustness. Models trained in distributed environments can be vulnerable to poisoning or evasion attacks. An attacker could, for example, inject manipulated data into one of the participating nodes, degrading the performance of the entire federated system without being easily detected. The development of autonomous systems requires integrating trust and resilience into their learning processes.

Ethical concerns, together with regulatory compliance requirements, are essential factors. GDPR [69] requires DAI systems to follow data minimisation principles and provide transparency and explainable operations. Ensuring that decentralised models do not violate users’ privacy, especially in systems that continuously learn from personal or behavioural data, is not a trivial task.

The interpretability of DAI decisions remains an open question. The decision-making process among multiple agents or nodes makes it challenging to identify the specific reason for an anomaly flag or the device that triggered a security response. The lack of transparency in these systems makes it difficult to establish trust, especially in critical sectors such as healthcare and energy.

Standardisation and interoperability remain significant obstacles for the field. The development of standalone prototypes for DAI solutions creates challenges for their integration into larger ecosystems. Future research should establish standardised communication interfaces, security frameworks, and performance benchmarks to support the real-world deployment of DAI solutions.

Across the reviewed literature, several recurring challenges emerge. These include decentralisation overhead, scalability constraints, trust management issues, interoperability limitations, and the persistent gap between theoretical promise and real-world deployment. Highlighting these shared concerns provides a clearer understanding of the current state of the field and the practical barriers that must be addressed for DAI-based IoT security to mature further.

Real-world deployment on resource-constrained IoT devices remains a key limitation for many DAI-based security approaches. In practice, the suitability of a method depends not only on its detection or coordination capability but also on its computational footprint, memory requirements, energy consumption, communication overhead, and latency profile. FL can be well-suited to edge environments because model training is distributed, but synchronisation costs and repeated communication rounds may still constrain its performance. MAS can support local autonomy and fast response, yet their effectiveness depends on the complexity of coordination among agents. DDL is typically the most demanding in terms of computation and memory, which can limit its feasibility on low-power devices unless it is combined with edge/cloud offloading or lightweight model designs. Accordingly, the practical deployment of DAI in IoT security requires a careful match between the security task, the threat model, and the available device resources.

Overall, achieving the full potential of DAI as a trusted and scalable IoT security approach depends on addressing these technical, operational, and regulatory challenges.

Future research should prioritise explainable DAI models, robust defences against poisoning and evasion attacks, interoperable deployment frameworks, and common benchmark datasets for fair comparison. Another important direction is the validation of DAI-based security methods in real-world heterogeneous IoT environments, especially in healthcare, industrial systems, and smart cities.

Also, future research should focus on addressing the practical and methodological limitations identified in this review. Promising directions include tighter integration of DAI with edge computing, stronger alignment with trustworthy AI principles, and the development of architectures that improve scalability, adaptability, and robustness in deployment. Further work is also needed to bridge the gap between conceptual advances and operational feasibility in real-world IoT environments.

In addition to all this, new trends are emerging, including the development of quantum computers, innovative security algorithms, and artificial intelligence-based protection systems. Quantum computers may be able to break current encryption in the future, so work is underway on algorithms that are resistant to such threats. In turn, combining DAI with quantum computing capabilities can enable the real-time handling of vast amounts of data.

## 7. Conclusions

This paper provided a structured review of DAI-based approaches to IoT security, with emphasis on security function, threat context, and deployment constraints. The reviewed literature indicates that DAI is most strongly supported in intrusion detection, anomaly detection, secure data processing, and collaborative defence, with Federated Learning and multi-agent approaches emerging as the most prominent and practically relevant directions. These methods offer clear advantages in distributed and privacy-sensitive environments, particularly where local decision-making and reduced dependence on centralised control are important.

Overall, the reviewed literature suggests strong potential for DAI-based IoT security, but the evidence base remains uneven in quality and maturity. Future claims about deployment readiness should therefore be grounded in studies that provide stronger validation, clearer reporting, and more realistic evaluation settings.

However, the review also shows that current research remains limited by resource constraints, communication overhead, adversarial vulnerability, interoperability challenges, and insufficient real-world validation. Future work should therefore prioritise lightweight and explainable designs, stronger robustness against poisoning and evasion attacks, improved benchmarking practices, and deployment-oriented evaluation in heterogeneous IoT environments. In this sense, DAI represents a credible and increasingly important direction for IoT security, but its long-term value will depend on how effectively these practical limitations are addressed.

## Figures and Tables

**Table 1:** Typical IoT attacks, their descriptions, and solutions.

Attack	Description and Solution	Ref.
Eavesdropping	Interception of Data During Transmission. Encryption (for example, TLS) helps prevent unauthorised access.	[55]
Side-Channel	Exploits physical characteristics like power or EM emissions. Mitigation includes masking and secure hardware design.	[56]
Firmware Tampering	Altering device firmware to insert backdoors. Secure boot and firmware signing reduce risk.	[57]
Data Injection	Injects false data to manipulate behaviour. Input validation and strong authentication help mitigate this.	[58]
Brute-Force	Attempts to guess credentials. Lockout policies and rate limiting are common defences.	[59]
Ransomware	Locks devices or data for ransom. Backups and updated defences are key countermeasures.	[60]
Botnets	Devices hijacked for DDoS attacks. Segmenting networks and updating firmware are helpful.	[61]
Physical Attacks	Direct Manipulation or Theft of Devices. Use tamper-evident hardware and secure enclosures.	[62]
Weak Credentials	Exploits default or weak passwords. Enforce strong policies and disable defaults.	[63]
Code Injection	Executes unauthorised code. Prevent with secure coding and input validation.	[64]
Backdoors	Exploits hidden or accidental access paths. Regular audits and penetration testing are essential.	[65]
Buffer Overflow	Exploits memory errors to crash or hijack systems. Use bounds checking and safe compilers.	[66]
Sensor Spoofing	Feeds false data to sensors. Use validation and multi-source comparison.	[67]
Privilege Escalation	Gains unauthorised high-level access. Apply the least privilege and update regularly.	[68]

**Table 2:** Summary of intrusion detection systems based on DAI methods.

Ref.	DAI Methods	Characteristic
[70]	K-means, K-nearest neighbour algorithms	• Identifies vulnerabilities in Cloud IoT environments.
[71]	Own solution	• Unsupervised IDS for WSN.
[72]	FL	• Uses autoencoders, variational autoencoders and adversarial autoencoders. • Intrusion and anomaly detection.
[73]	BHO	• Cooperative SDN-based IDS for IoT using BHO feature selection and CNN-based detection.
[75]	FL, Differential Privacy	• Ability to predict and determine attack nature, recommended for privacy-critical environments like medical and financial domains.
[77]	CNN	• Uses CNN for industrial data classification (Industry 4.0). • Utilizes ITrustFootnote1 platform for data collection.
[78]	ML	• Blockchain-Based Collaborative Distributed Intrusion Detection System for Vehicle Sharing Networks.
[81]	CNN, LSTM	• Distributed DL intrusion detection method using Apache Spark framework for Internet of Vehicles.
[85]	DRNN	• Suitable for limited resource IoT networks due to enhanced generalisation and decentralised nature.
[86]	FL-based own solution	• Blockchain-Based Attack Detection Algorithm using end-to-end clustering algorithm.
[87]	FL	• Combats poisoning attacks against the FL-enabled IDS on heterogeneous IoT data.
[88]	FL	• Utilizes a new loss function for local model training.
[89]	FL	• Monitors and analyses medical data in decentralized environments. • Uses RN to learn latent relationships of medical data.
[90]	FL	• Model outperforms existing federated DL methods in IoT anomaly detection.
[91]	FL	• Utilizes local learning for data privacy. • Enhances detection model through model updates with an aggregate server. • Mitigates attacks against agricultural IoTs.

**Table 3:** Summary of specific attack detection systems based on DAI methods.

Ref.	DAI Methods	Characteristic
[92]	SI	• Detects jamming, denial of sleep, manipulation, and cheating in IEEE 802.15.4-based WSNs; based on simulation results.
[97]	Multi-agent network	• Detects malware attacks on IoT devices.
[98]	FL	• Detects malware attacks on IoT device.
[99]	ML	• Detects DDoS attacks in blockchain-based IoT solutions.
[100]	FFNN, LSTM	• Detects DDoS attacks.
[101]	ANN	• Detects DDoS attacks in software-defined networking IoT solutions.
[102]	GAN, FL	• Detects poisoning attack in social IoT.
[104]	FFNN, LSTM	• Detects DDoS in IoT Fog computing architectures.
[105]	FL	• Detects DDoS attacks.

**Table 4:** Summary of secure data processing systems based on DAI methods.

Ref.	DAI Methods	Characteristic
[106]	Multi-agent multi-armed bandit model	• Utility-aware caching for IoT Edge Computing. • Multiple edge servers employ multi-agent RN to collectively determine the optimal caching strategy to maximise the overall long-term value of the system.
[108]	ML	• Solution for IoT Cloud Computing. • Utilises data obtained from edge devices to implement a distributed ML model and achieve prompt response at the edge.
[109]	Evolutionary algorithms, SI	• Edge-computing architecture for large-scale IoT-based cyber-physical systems that use distributed multi-task learning over edge-computing networks.
[110]	FL	• Actor model to train neural networks on fog devices in an IoT setting.
[112]	CNN, DRN	• Mechanism for adjusting radio settings of end devices during operation. • Capable of accommodating various application requirements simultaneously and adapting to network changes.
[114]	CNN, DRN	• Distribution CNN execution on edge servers to minimise system delay and energy consumption.
[117]	FL	• Enhances security in smart cities by addressing privacy disclosure attacks.
[118]	FL	• Protects data privacy and enables personalised training of non-independent, identically distributed data on IoT devices.

**Table 5:** Summary of other security solutions using DAI methods.

Ref.	DAI Methods	Characteristic
[123]	Own solution	• Algorithm for the mobile human position prediction based on Particle Swarm Optimisation and Elman neural network.
[127]	FL	• Preserves privacy by keeping data on-device and aggregating local updates for botnet detection.
[128]	DL	• Distributed public key infrastructures incorporating key generation engines and DL algorithms. • Utilises DL algorithms to analyse secure datasets and detect malicious activities within certificate logs.
[134]	FL	• Secures against cyberattacks in digital twin-based industrial IoT environments.
[135]	FL	• Offloads and schedules healthcare systems in blockchain-based networks.
[136]	Occupy-Interference Mitigation	• Anti-interference optimization scheme for IoT-edge system.
[138]	FL	• Architecture of the IoT application is designed based on microservices, which enables it to be organised as a set of small, independent and reusable components.

**Table 6:** Overview of DAI techniques vs. IoT threat types.

Attack Type	FL	Multi-Agent	DL	Blockchain	SI
DDoS	well established	moderately supported	well established	moderately supported	not reported
Ransomware	moderately supported	experimental	moderately supported	well established	not reported
Insider Threat	not reported	well established	not reported	well established	experimental
Poisoning	well established	not reported	moderately supported	experimental	not reported
Spoofing/Replay	experimental	moderately supported	not reported	moderately supported	moderately supported

## Data Availability

No new data were created or analyzed in this study. Data sharing is not applicable.

## Abbreviations

The following abbreviations are used in this manuscript:

AI	Artificial Intelligence
BHO	Black Hole Optimization algorithm
CNN	Convolutional Neural Network
CIA triad	Confidentiality, Integrity, and Availability triad
DAI	Distributed Artificial Intelligence
DDL	Distributed Deep Learning
(D)DoS	(Distributed) Denial of Service attack
DRN	Deep Reinforcement Learning
DRNN	Dense Random Neural Network
FFNN	Feed-Forward Neural Network
FL	Federated Learning
GAN	Generative Adversarial Network
GDPR	General Data Protection Regulation
IDS	Intrusion Detection System
IPS	Intrusion Prevention System
IoT	Internet of Things
JDK	Java Development Kit
LSTM	Long Short-Term Memory
MAS	Multi-Agent Systems
ML	Machine Learning
MQTT	Message Queuing Telemetry Transport
NFC	Near Field Communication
PCNN	Parallel Convolutional Neural Network
PSD	Positive Semi-Definite
RL	Reinforcement Learning
SDN	Software-Defined Networking
SI	Swarm Intelligence
TLS	Transport Layer Security
WSN	Wireless Sensor Networks
